# Relationship Between Self-Efficacy and Burnout Syndrome in Resident Physicians in Metropolitan Lima

**DOI:** 10.3390/ijerph23050679

**Published:** 2026-05-20

**Authors:** Rafael Emiliano Sulca Quispe, Danny Vergel Moncada, Filomeno Teodoro Jauregui Francia

**Affiliations:** 1 Faculty of Education, Universidad Nacional Federico Villarreal, Lima 15088, Peru; 2 Hospital de Lima Este-Vitarte, Ate 15491, Peru; davermon46@gmail.com; 3 Faculty of Medicine, Universidad Ricardo Palma, Lima 15039, Peru; filomeno.jauregui@urp.edu.pe

**Keywords:** self-efficacy, burnout syndrome, resident physicians, occupational health, medical residency, mental health

## Abstract

**Highlights:**

**Public health relevance—How does this work relate to a public health issue?**

- Burnout syndrome is a common problem among medical trainees and compromises their physical and emotional well-being.
- Identifying protective factors, such as self-efficacy, is relevant to occupational health and quality of care in hospitals.

**Public health significance—Why is this work of significance to public health?**

- The study provides local evidence on the relationship between self-efficacy and burnout in resident physicians in Metropolitan Lima.
- The findings show that higher self-efficacy is associated with less emotional exhaustion, less depersonalization, and greater personal accomplishment.

**Public health implications—What are the key implications or messages for practitioners, policy makers and/or researchers in public health?**

- Strengthening self-efficacy during residency could reduce professional burnout and promote the psychological well-being of the resident.
- Mentoring programs, emotional support, and burnout prevention should be considered priority institutional strategies.

**Abstract:**

Burnout syndrome is one of the main problems among healthcare personnel. In resident physicians, this syndrome can affect both their clinical performance and their personal well-being. Objective: To determine the relationship between self-efficacy and burnout syndrome in resident physicians at hospitals in Metropolitan Lima. Method: An observational, analytical, cross-sectional study was conducted. The sample consisted of 521 resident physicians from different specialties, selected using non-probability sampling. The General Self-Efficacy Scale and the Maslach Burnout Inventory were used. Results: Self-efficacy was inversely correlated with emotional exhaustion (rho = −0.387; *p* < 0.001), depersonalization (rho = −0.347; *p* < 0.001), and global burnout syndrome (rho = −0.453; *p* < 0.001), and directly correlated with personal accomplishment (rho = 0.530; *p* < 0.001). The structural equation model showed an excellent fit and confirmed a direct effect of self-efficacy on the dimensions of burnout. Conclusions: Higher levels of self-efficacy are associated with lower levels of emotional exhaustion, depersonalization, and overall burnout, as well as greater personal accomplishment in resident physicians.

## 1. Introduction

Medical practice is an exhausting task, especially for resident physicians, who face long working hours, staff shortages, and high emotional pressure in their medical activities [1,2]. In this sense, two concepts have become relevant: self-efficacy, which is confidence in one’s personal ability to face specific challenges [3], and burnout syndrome (BS), a disorder related to physical and emotional exhaustion, which is accompanied by depersonalization and a reduced perception of accomplishment [4].

Burnout syndrome is not a new problem in the medical field, but its increase is especially alarming [5]. Several studies conducted during the pandemic reported that between 30% and more than half of healthcare personnel experienced burnout [6]. This condition negatively affects both the well-being of healthcare professionals and the quality of care and patient safety [7,8]. Research in Thailand shows that the prevalence of burnout is highest during medical residency training [9].

Self-efficacy emerges as an essential resource for coping with these adversities [8]. Physicians with high levels of self-efficacy tend to perceive work challenges as opportunities rather than threats, enabling them to better manage stress and prevent burnout [9]. Conversely, those with limited self-efficacy face greater difficulties in dealing with daily demands, increasing their vulnerability to burnout [10].

In the international context, according to Al-Huseini et al. [11] burnout in resident physicians has a prevalence of 25.8%, with high levels of emotional exhaustion and depersonalization. Furthermore, age and marital status are identified as associated factors. The relationship between self-efficacy and burnout syndrome in resident physicians is a widely discussed topic. In China, Du et al. [12] conducted a study in pediatric resident physicians, which demonstrated that self-efficacy is significantly related to burnout and, moreover, can positively predict burnout and its dimensions through resilience. Similarly, Li et al. [13] found that Chinese resident physicians with lower self-efficacy tend to experience emotional distress and burnout. In the US, Ju et al. [14] reported that high levels of self-efficacy were associated with low levels of burnout in residents who worked during the pandemic. Meanwhile, Erschens et al. [15] demonstrated that self-efficacy has a protective effect against burnout in German physicians in training. Tipwong et al. [16] in Thailand found that higher clinical self-efficacy significantly predicts lower burnout levels in physicians.

In the national context, very few studies have linked self-efficacy with burnout, despite the unfavorable work environments faced by resident physicians [17,18]. In Peru, being a resident physician is considered a risk factor for developing burnout, which negatively impacts their mental health and patient care [19,20]. On the other hand, professional self-efficacy has been identified as a key psychological resource in medical performance, associated with better clinical decision-making and a lower probability of iatrogenic events in the hospital setting [21,22].

This study is justified by its significant theoretical implications, as it will elucidate the role of self-efficacy as a psychological construct influencing human behavior, based on Bandura’s social learning theory [23]. Furthermore, regarding burnout syndrome, it is grounded in the theoretical model developed by Maslach, which explains its dimensions and impact in the workplace [24]. From a clinical perspective, this is relevant because burnout affects the performance of resident physicians and can indirectly impact patient safety. Finally, in terms of administrative implications, both burnout syndrome and low levels of self-efficacy can lead to absenteeism and decreased productivity, necessitating the implementation of training programs and institutional stress management strategies, which entails a greater commitment of financial resources.

Therefore, this article poses the following research question: What is the relationship between self-efficacy and burnout syndrome in resident physicians at hospitals in Metropolitan Lima? Likewise, the objective was to determine the relationship between self-efficacy and burnout syndrome in resident physicians at hospitals in Metropolitan Lima.

## 2. Materials and Methods

### 2.1. Study Design

A quantitative, cross-sectional study was conducted. Correlational cross-sectional studies allow for the analysis of associations between variables such as psychosocial factors and clinical or occupational aspects, which are widely used in the field of health research [25].

### 2.2. Population and Sample

The study population consisted of 824 resident physicians from the Lima Este-Vitarte Hospital and the Medical Residency and Specialization School of the Faculty of Medicine at Ricardo Palma University, located in hospitals throughout Metropolitan Lima. The sample size was calculated using the formula for finite populations, resulting in a minimum size of 262 participants. However, data were collected from 521 resident physicians, selected using non-probability convenience sampling. Non-probability convenience sampling selects accessible subjects who meet the inclusion criteria defined by the researchers [26].

### 2.3. Inclusion and Exclusion Criteria

Inclusion criteria:

- Doctors in the first, second, and third year of medical residency.
- Doctors over 18 years of age.
- Doctors who declared their consent to participate in the research.

Exclusion criteria:

- Resident physicians diagnosed with anxiety and depression.
- Resident doctors resigning from the program.
- Medical residents on medical leave.
- Resident doctors on vacation.

### 2.4. Instruments

The General Self-Efficacy Scale (GSES) developed by Schwarzer and Jerusalem [27] was used, which consists of 10 items on a 4-point Likert scale. This scale aims to evaluate the perceived ability to cope with difficult situations. It has shown adequate psychometric properties in international populations and in health professionals, with reliability coefficients greater than 0.80 [28]. The instrument is unidirectional. 

The Maslach Burnout Inventory [29], consisting of 22 items differentiated into three dimensions—emotional exhaustion, depersonalization, and personal accomplishment—was used to assess burnout syndrome. In a recent Peruvian study of health professionals, α = 0.886 and ω = 0.883 were reported for emotional exhaustion, α = 0.848 and ω = 0.802 for personal accomplishment, and α = 0.574 and ω = 0.492 for depersonalization [30].

The instruments were administered in the Spanish language, duly validated and with their respective reliability.

### 2.5. Procedure and Data Analysis

Data were collected after obtaining informed consent, using self-administered questionnaires distributed online via Google Forms. Resident physicians were invited to complete the instruments through the Medical Residency School of a private university and a public hospital in Metropolitan Lima. Anonymity, confidentiality, and voluntary participation were guaranteed. Regarding data analysis, frequencies, percentages, and measures of central tendency were calculated to describe sociodemographic characteristics and burnout levels. Subsequently, Spearman’s rank correlation coefficient was used due to the non-normal distribution of the data, with the support of IBM SPSS Statistics version 26.0 (IBM Corp., Armonk, NY, USA).

A structural equation model (SEM) was designed to evaluate the association between self-efficacy and burnout syndrome using RStudio version 2025.05.1+513 (Posit Software, PBC, Boston, MA, USA). The SEM was estimated using robust maximum likelihood estimation (BLL). Prior to this, the assumption of normality was assessed using Kolmogorov–Smirnov tests and skewness and kurtosis analyses, revealing a non-normal distribution for some variables, thus justifying the use of a robust estimator. The model included latent variables corresponding to self-efficacy (SELFEFFICACY) and the dimensions of burnout syndrome, emotional exhaustion (EE), depersonalization (D), and personal accomplishment (PA), which were measured through their respective observed indicators.

Regarding the sample size, 521 participants were included, which is adequate for the SEM analysis, considering the recommendations that suggest a minimum of 200 cases or a ratio of at least 10 subjects per estimated parameter, thus ensuring the stability and precision of the estimators. Likewise, the model assumptions were verified, including the absence of multicollinearity, linearity between variables, and the adequate specification of the theoretical model. The quality of fit was assessed using standard indices such as the chi-square (χ^2^) test, the comparative fit index (CFI), the Tucker–Lewis index (TLI), and the root mean square error of approximation (RMSEA), following criteria accepted in the specialized literature.

### 2.6. Ethical Aspects

The study was approved by the Institutional Research Ethics Committee of the Lima Este-Vitarte Hospital, approval code No. 082-2025-CIE/HLEV. All participants were informed about the study objectives and provided their informed consent voluntarily. The confidentiality and anonymity of the collected information were also guaranteed.

## 3. Results

### 3.1. Descriptive Analysis

The sample consisted of 521 resident physicians. Participants aged 31 to 40 years predominated (57.01%), followed by those aged 30 years or younger (29.17%) and those aged 41 years or older (13.82%). Regarding gender, a higher proportion of women (56.60%) was observed compared to men (43.40%). As for marital status, the most frequent group was single (60.3%), followed by single with a partner (19.2%), cohabiting (9.4%), married (8.1%), and divorced (3.1%). According to the training site, the majority belonged to MINSA (Ministry of Health) facilities (65.07%), followed by EsSalud (Social Security) (31.29%) and private clinics (3.65%). Regarding specialty, the group of residents from non-surgical areas predominated (64.1%), compared to surgical specialties (35.9%) (Table 1).

When analyzing the distribution of burnout dimensions by sex and specialty, emotional exhaustion was more frequent in women (43.1%) than in men (36.7%), and in surgical specialties (43.6%) compared to non-surgical specialties (33.1%). Depersonalization was slightly more frequent in men (40.3%) than in women (38.3%), and more frequent in surgical specialties (44.6%) than in non-surgical specialties (31.5%). Global burnout syndrome was present in more than half of the women (56.9%) and in 52.2% of the men; by specialty, it was more frequent in surgical residents (58.8%) than in non-surgical residents (46.7%). According to the classification shown in the table, the reported frequency for burnout was higher in women (21.4%) than in men (19.5%) and in surgical specialties (23.0%) compared to non-surgical specialties (16.4%) (Table 2).

Regarding burnout levels, emotional exhaustion showed a significant distribution between low (43.8%) and high (40.3%) levels, while only 15.9% were at the medium level. Depersonalization presented a similar pattern, with a predominance at the low level (45.3%) and a high proportion at the high level (39.2%). Personal accomplishment, meanwhile, was concentrated mainly at the high level (49.1%), followed by the medium (27.3%) and low (23.6%) levels. Taken together, these findings demonstrate that, even though some residents maintain favorable levels of personal accomplishment, a significant burden of emotional exhaustion and depersonalization persists in a considerable portion of the sample (Table 3).

### 3.2. Inferential Analysis

Inferential analysis showed that self-efficacy was significantly related to all dimensions of burnout. A negative correlation was found between self-efficacy and emotional exhaustion (rho = −0.387; *p* < 0.001), as well as between self-efficacy and depersonalization (rho = −0.347; *p* < 0.001). In contrast, the relationship between self-efficacy and personal accomplishment was positive and of greater magnitude (rho = 0.530; *p* < 0.001). Furthermore, self-efficacy was negatively correlated with global burnout syndrome (rho = −0.453; *p* < 0.001). Interpretively, these results indicate that higher self-efficacy is associated with lower levels of emotional exhaustion, depersonalization, and global burnout, and with greater personal accomplishment. It should be noted that the context of the hospitals will differentiate these results, given that some hospitals are part of the Ministry of Health (MINSA) and EsSalud, which characterizes the Peruvian health system as segmented and fragmented (Table 4).

**Figure 1:**
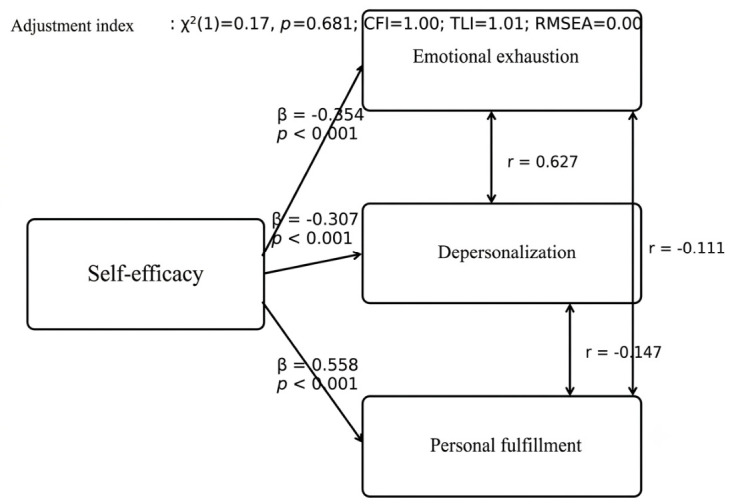
Structural Equation Model (SEM).

A structural model was estimated in which self-efficacy directly predicted the three dimensions of burnout, allowing for covariance between them. This model showed an excellent fit: χ^2^(1) = 0.17, *p* = 0.681; CFI = 1.00; TLI = 1.01; RMSEA = 0.00. Self-efficacy was negatively associated with emotional exhaustion (β = −0.354, *p* < 0.001) and depersonalization (β = −0.307, *p* < 0.001), and positively associated with personal accomplishment (β = 0.558, *p* < 0.001). These findings indicate that higher levels of self-efficacy are associated with less emotional distress and a greater perception of professional accomplishment among resident physicians (Figure 1).

## 4. Discussion

This study demonstrated that self-efficacy is significantly related to burnout syndrome in resident physicians at hospitals in Metropolitan Lima. Specifically, an inverse correlation was found between self-efficacy and the dimensions of emotional exhaustion and depersonalization, as well as a direct correlation with personal accomplishment. Furthermore, self-efficacy was negatively associated with overall burnout syndrome. These findings indicate that a greater perceived capacity to successfully cope with the demands of the professional environment is associated with a lower prevalence of occupational burnout, thus supporting the theoretical hypothesis proposed in this research.

The results obtained are consistent with previous evidence reported in resident physicians. Du et al. found that self-efficacy is significantly related to burnout and its dimensions in pediatric residents, and also plays a role in explaining it through resilience [11]. Similarly, Li et al. reported that lower levels of self-efficacy are associated with greater emotional distress and burnout in Chinese residents [12]. Along the same lines, Ju et al. reported that residents with high levels of self-efficacy had lower levels of burnout during the pandemic [13]. Likewise, Erschens et al. indicated that self-efficacy has a protective effect against burnout in physicians in training [14], while Tipwong et al. described that higher clinical self-efficacy predicts lower levels of burnout and greater professional fulfillment [15]. Taken together, this background research supports the consistency of the findings of the present study.

These results can be understood in light of the role of self-efficacy as a psychological coping resource. Resident physicians with higher self-efficacy tend to interpret the clinical, academic, and emotional demands of residency as manageable challenges, rather than uncontrollable threats, thus favoring more adaptive responses to stress. Consequently, this could reduce the likelihood of developing emotional exhaustion and depersonalization, while simultaneously strengthening their perception of competence and professional achievement. This interpretation is consistent with the points made in the study’s introduction, which suggest that self-efficacy allows for better coping with work pressure and reduces vulnerability to burnout [8,9,10].

The descriptive analysis also revealed a significant burden of burnout in the studied population. More than half of the resident physicians presented with burnout syndrome, and high rates of emotional exhaustion and depersonalization were observed. This finding confirms that residency is a context of high demands and psychosocial risk. The literature had already noted that burnout is a frequent problem among healthcare professionals and that its prevalence can increase significantly during medical residency [4,7]. Furthermore, it has been described that this condition negatively affects both the professional’s well-being and the quality of care and patient safety [5,6], so its presence in this population should not be interpreted as an isolated individual phenomenon, but rather as a problem of institutional and educational relevance.

Regarding the characteristics of the subgroups evaluated, a higher frequency of emotional exhaustion and overall burnout was observed in women and in residents of surgical specialties. Although the cross-sectional design does not allow for establishing causal relationships, this trend could be linked to differences in the intensity of the workload, the type of clinical pressure, and the conditions of clinical training. In surgical specialties, for example, the high technical demands, the need for immediate response, and the pressure to perform could increase the risk of emotional exhaustion. Similarly, female residents may be exposed to a more complex set of academic, professional, and personal demands, which could help explain the higher frequency observed. These findings are plausible and consistent with the evidence that recognizes residency as a particularly vulnerable stage for physicians’ mental health [17,18].

A particularly important contribution of this study was the incorporation of structural equation modeling. This analysis showed an excellent fit and confirmed that self-efficacy has a direct effect on the dimensions of burnout: negatively on emotional exhaustion and depersonalization, and positively on personal accomplishment. This result strengthens the interpretation of the phenomenon, as it not only demonstrates significant bivariate associations but also reveals a coherent relational structure among the variables studied. Methodologically, this lends greater robustness to the study’s central hypothesis, and in practical terms, it suggests that self-efficacy could be a strategic variable for designing preventive interventions aimed at promoting the psychological well-being of residents.

In the national context, evidence on the relationship between self-efficacy and burnout in resident physicians remains scarce, despite the importance of the problem and the unfavorable working conditions this group faces [16]. Therefore, the present study provides relevant evidence for the Peruvian context and helps fill a gap in the local literature. Furthermore, these results are particularly important considering that professional self-efficacy has also been linked to better clinical decision-making and a lower risk of iatrogenic events in the hospital setting [19,20]. In this sense, strengthening self-efficacy could not only benefit the resident’s mental health but also their professional performance and patient safety.

The clinical implications of the findings in this research—emotional exhaustion, depersonalization, and low personal accomplishment—are evident in the participants affected by burnout syndrome. From a practical perspective, the study’s findings suggest the need to implement institutional strategies aimed at strengthening personal resources and preventing professional burnout during residency. These strategies could include mentoring programs, psychological support, formative clinical supervision, spaces for constructive feedback, and stress management training. These interventions would be particularly relevant in hospital settings where the workload and academic demands are high and sustained. Thus, strengthening self-efficacy could constitute not only a measure for promoting mental health but also a strategy for improving the quality of training in medical residency. Furthermore, the theoretical implications of the self-efficacy construct as an explanatory model for burnout have been corroborated. Finally, administratively, low personal accomplishment has translated into absenteeism and resignation from medical residency.

However, the results should be interpreted in light of certain limitations. The non-probability sampling limits the extrapolation of the findings to all resident physicians in Metropolitan Lima. The use of self-administered instruments may introduce self-reporting bias or social desirability bias. Furthermore, the participants’ year of residency, number of on-call hours, clinical workload, and possible pre-existing mental health disorders warrant further research to address these limitations. Despite these, the study has significant strengths, such as the sample size achieved, the inclusion of a population highly relevant to occupational health and medical education, and the use of robust statistical analyses that included descriptive and correlational statistics, as well as structural modeling.

## 5. Conclusions

- Self-efficacy was significantly and inversely related to burnout syndrome in resident physicians at hospitals in Metropolitan Lima. Higher levels of self-efficacy were associated with lower levels of emotional exhaustion, depersonalization, and overall burnout, as well as greater personal accomplishment.
- The descriptive analysis revealed a significant burden of burnout in the studied population, with emotional exhaustion and depersonalization predominating in a considerable proportion of residents. Furthermore, women and residents in surgical specialties showed higher rates of emotional exhaustion and overall burnout, suggesting potentially more vulnerable subgroups.
- Structural equation modeling confirmed that self-efficacy has a direct effect on the dimensions of burnout, solidifying its role as a protective psychological resource in the context of medical residency. Consequently, it is recommended that institutional strategies be incorporated to strengthen self-efficacy, clinical mentoring, emotional support, and the prevention of burnout during specialist training.
- Finally, it is suggested that future research develop longitudinal and probabilistic designs in order to assess the temporal stability of these associations and to delve deeper into the mechanisms by which self-efficacy influences the occupational mental health of resident physicians.

## Figures and Tables

**Table 1:** Sociodemographic data.

Sociodemographic Data	*n*	%
Age		
≤30 years	152	29.17%
31–40 years	297	57.01%
≥41 years	72	13.82%
Gender		
Female	295	56.60%
Male	226	43.40%
Marital Status		
Single	314	60.3%
Single with partner	100	19.2%
Cohabitant	49	9.4%
Married	42	8.1%
Divorced	16	3.1%
Headquarters		
MINSA	339	65.07%
EsSalud	163	31.29%
Private clinic	19	3.65%
Specialty		
Surgical	204	35.9%
Non-Surgical	317	64.1%

**Table 2:** Distribution of burnout dimensions according to sex and specialty.

	Gender				Specialty			
	Male		Female		Surgical		Non-Surgical	
	*n*	%	*n*	%	*n*	%	*n*	%
EA	83	36.7	127	43.1	89	43.6	105	33.1
D	91	40.3	113	38.3	91	44.6	100	31.5
RP	44	19.5	63	21.4	47	23.0	52	16.4
SB	118	52.2	168	56.9	120	58.8	148	46.7

Note: SB: Burnout syndrome EA: Emotional exhaustion; D: Depersonalization; RP: Personal accomplishment.

**Table 3:** Burnout levels.

	Low		Half		High	
	*n*	%	*n*	%	*n*	%
EA	228	43.8%	83	15.9%	210	40.3%
D	236	45.3%	81	15.5%	204	39.2%
RP	123	23.6%	142	27.3%	256	49.1%

Note: EA: Emotional exhaustion; D: Depersonalization; RP: Personal accomplishment.

**Table 4:** Rho correlations between self-efficacy and burnout dimensions.

	EA	D	RP	SB
Self-efficacy	−0.387	−0.347	0.530	−0.453
Sign (bilateral)	0.000	0.000	0.000	0.000
N	521	521	521	521

Note: EA: Emotional exhaustion; D: Depersonalization; RP: Personal accomplishment; SB: Burnout syndrome.

## Data Availability

The data presented in this study are available upon reasonable request to the corresponding author, subject to ethical and confidentiality restrictions.

## Abbreviations

EA	Emotional exhaustion
D	Depersonalization
RP	Personal fulfillment
SB	Burnout syndrome
GSES	General Self-Efficacy Scale
MBI	Maslach Burnout Inventory

## References

[1] Surawattanasakul V., Siviroj P., Kiratipaisarl W. (2024). Resident physician burnout and association with working conditions, psychiatric determinants, and medical errors: A cross-sectional study. PLoS ONE.

[2] Fujikawa H., Tamune H., Nishizaki Y., Nagasaki K., Kobayashi H., Nojima M., Sekine M., Shimizu T., Yamamoto Y., Shikino K. (2025). Associations between patient care ownership, burnout, and job satisfaction among medical residents: A nationwide cross-sectional study in Japan. Sci. Rep..

[3] Yuen B., Asmar A., Kibble J. (2025). Trainee Identification of the Self-Efficacy Domains Needed to Succeed in Undergraduate and Graduate Medical Education. Med. Sci. Educ..

[4] Bordbar S., Mousavi S.M., Samadi S. (2025). The association between burnout and medical professionalism in medical trainees: A systematic review. BMC Med. Educ..

[5] Ng I.K.S., Tham S.Z.J., Chong K.M., Goh G.W., Thong C., Teo K.S.H. (2025). Burnout among medical residents: Key drivers and practical mitigating strategies. Postgrad. Med. J..

[6] Dūdina K., Martinsone B. (2025). Psychosocial Risks and Protective Factors for Healthcare Worker Burnout During the Post-Acute Phase of the COVID-19 Pandemic. Eur. J. Investig. Health Psychol. Educ..

[7] Daneshvar E., Otterbach S. (2025). Workplace stressors and burnout among healthcare professionals: Pandemic perspectives and implications for future public health crises. Sci. Rep..

[8] Hueto Madrid J.A., Hargreaves J., Buchelt B. (2025). Putting patients at risk: The effect of healthcare professional burnout on patient care in the operating room: A narrative review. J. Patient Saf..

[9] Surawattanasakul V., Siviroj P., Kiratipaisarl W., Sirikul W., Phetsayanavin V., Pholvivat C., Auernaruemonsuk N., Lamlert C. (2025). Physician burnout, associated factors, and their effects on work performance throughout first-year internships during the COVID-19 pandemic in Thailand: A cross-sectional study. BMC Public Health.

[10] Chen H., Cao Z., Zhang X., Duan H., Jiang S., Cai C. (2025). The association between perceived stress and resilience among medical staff during public health emergencies: Mediating effect of self-efficacy. BMC Psychol..

[11] Al-Huseini S., Al Alawi M., Al-Balushi N., Al Sinawi H., Mirza H., Al Balushi R., Al Balushi M., Jose S., Cucchi A., Al-Sibani N. (2025). Prevalence and predictors of occupational burnout among first-year medical residents in Oman: The role of trait emotional intelligence. BJPsych Int..

[12] Du Y., Qiao L., Dong L., Wan C., Yang X., Liu H.-M. (2024). The relationship between self-efficacy, resilience and burnout in pediatric residents: A cross-sectional study in western China. BMC Med. Educ..

[13] Li Z., Wu M., Zhang X., Yan K., Wang X., Xu H., Li P., Liu Y., Deng Q., Li X. (2024). Interrelationships of stress, burnout, anxiety, depression, quality of life and suicidality among Chinese residents under standardized residency training: A network analysis. Ann. Med..

[14] Ju T., Mikrut E.E., Spinelli A., Romain A.M.N., Brondolo E., Sundaram V., Pan C.X. (2022). Factors associated with burnout among resident physicians responding to the COVID-19 pandemic: A 2-month longitudinal observation study. Int. J. Environ. Public Health Res..

[15] Erschens R., Schröpel C., Herrmann-Werner A., Junne F., Listunova L., Heinzmann A., Keis O., Schüttpelz-Brauns K., Herpertz S.C., Kunz K. (2024). The mediating role of self-efficacy in the relationship between previous professional training and resilience to burnout in medical training: A multicenter cross-sectional study. BMC Med. Educ..

[16] Tipwong A., Hall N.C., Snell L., Chamnan P., Moreno M., Harley J.M. (2024). Clinical teaching self-efficacy positively predicts personal accomplishment and negatively predicts burnout among Thai physicians: A cross-sectional survey. BMC Med. Educ..

[17] Bernales-Turpo D., Quispe-Velasquez R., Flores-Ticona D., Saintila J., Mamani P.G.R., Huancahuire-Vega S., Morales-García M. (2022). Burnout, professional self-efficacy and life satisfaction as predictors of job performance in health workers: The mediating role of work engagement. Prim. Health Care Res. Dev..

[18] Villarreal-Zegarra D., Bernabé-Ortiz A., Carrillo-Larco R.M., Cabieses B., Blukacz A., Bellido-Boza L., Mezones-Holguin E. (2022). Relationship between job satisfaction, burnout syndrome and depressive symptoms among physicians in Peru: A cross-sectional study. BMJ Open.

[19] Nombera-Aznaran N., Bazalar-Palacios J., Nombera-Aznaran M., Rojas-Del-Aguila M., Aznaran-Torres R. (2025). Burnout syndrome and psychological workplace violence among Peruvian physicians: A cross-sectional study. BMC Health Serv. Res..

[20] Flores-Cohaila J.A., Estela-Fernandez C.A., Miranda-Chavez B., Flores-Arocutipa J.P., Lujan-Minaya J.C., Huarcaya-Victoria J., Copaja-Corzo C. (2026). The spectrum of burnout: An analysis of latent profiles in Peruvian resident physicians. Educ. Med..

[21] Fernandes C., Barros C., Baylina P. (2025). Burnout among healthcare workers: Insights for holistic well-being. Healthcare.

[22] Prazeres F., Santiago L.M., Simões J.A. (2025). Factors associated with family physicians’ perceived self-efficacy in managing patients with multimorbidity. BMC Fam. Pract..

[23] Honicke T., Broadbent J., Fuller-Tyszkiewicz M. (2023). The self-efficacy and academic performance reciprocal relationship: The influence of task difficulty and baseline achievement on learner trajectory. High. Educ. Res. Dev..

[24] Kadosh E., Alfasi Okounev R., Darienko D., Luxman Balasanov I., Mostafa Asali E., Naser Y., Taki Fdely M., Rozani V. (2025). Self-efficacy in clinical decision-making under stress and its association with resilience and anxiety in healthcare trainees. Nurse Educ..

[25] Setia M.S. (2022). Methodology series module 3: Cross-sectional studies. Indian J. Dermatol..

[26] Etikan I., Musa S.A., Alkassim R.S. (2016). Comparison of convenience sampling and purposive sampling. Am. J. Theor. Appl. Stat..

[27] Schwarzer R., Jerusalem M., Weinman J., Wright S., Johnston M. (1995). Generalized Self-Efficacy Scale. Measures in Health Psychology: A User’s Portfolio.

[28] Luszczynska A., Scholz U., Schwarzer R. (2005). The General Self-Efficacy Scale: Multicultural validation studies. J. Psychol..

[29] Maslach C., Jackson S.E., Leiter M.P. (2018). Maslach Burnout Inventory Manual.

[30] Yslado Méndez R.M., Sánchez-Broncano D., De La Cruz-Valdiviano C., Quiñones-Anaya I., Reynosa Navarro E. (2024). Psychometric properties of the Maslach Burnout Inventory in healthcare professionals, Ancash Region, Peru. F1000Research.

